# Intratumoral Genetic Heterogeneity in Papillary Thyroid Cancer: Occurrence and Clinical Significance

**DOI:** 10.3390/cancers12020383

**Published:** 2020-02-07

**Authors:** Laura Fugazzola, Marina Muzza, Gabriele Pogliaghi, Mario Vitale

**Affiliations:** 1 Division of Endocrine and Metabolic Diseases, IRCCS Istituto Auxologico Italiano, Milan and Department of Pathophysiology and Transplantation, University of Milan, 20149 Milan, Italy; marina.muzza@unimi.it (M.M.); gabriele.pogliaghi@studenti.unimi.it (G.P.); 2 Department of Medicine, Surgery and Dentistry, University of Salerno, Baronissi, 84081 Salerno, Italy

**Keywords:** heterogeneity, thyroid cancer, BRAF 3, RET/PTC, clonality

## Abstract

Intratumoral heterogeneity (ITH) refers to a subclonal genetic diversity observed within a tumor. ITH is the consequence of genetic instability and accumulation of genetic alterations, two mechanisms involved in the progression from an early tumor stage to a more aggressive cancer. While this process is widely accepted, the ITH of early stage papillary thyroid carcinoma (PTC) is debated. By different genetic analysis, several authors reported the frequent occurrence of PTCs composed of both tumor cells with and without *RET/PTC* or *BRAF*^V600E^ genetic alterations. While these data, and the report of discrepancies in the genetic pattern between metastases and the primary tumor, demonstrate the existence of ITH in PTC, its extension and biological significance is debated. The ITH takes on a great significance when involves oncogenes, such as *RET* rearrangements and *BRAF*^V600E^ as it calls into question their role of driver genes. ITH is also predicted to play a major clinical role as it could have a significant impact on prognosis and on the response to targeted therapy. In this review, we analyzed several data indicating that ITH is not a marginal event, occurring in PTC at any step of development, and suggesting the existence of unknown genetic or epigenetic alterations that still need to be identified.

## 1. Introduction

Genomic analysis of cancer samples reveals a complex mutational landscape with vast *intertumor* and *intratumoral heterogeneity*. *Intertumoral heterogeneity* refers to genetic and phenotypic variants occurring among individuals with the same tumor type. *Intratumoral heterogeneity (ITH)* refers to a subclonal diversity that may be observed within a tumor lesion. Intratumoral genetic heterogeneity is a paradigm of carcinogenesis, a process that transforms a tumor into a more aggressive cancer through gain of genetic alterations. This process transforms a clonal neoplasm into a mass of genetically different subclones that may be intermixed or spatially separated within the neoplastic tissue. Tumor subclones are characterized by differential gene expression due to both genetic and epigenetic heterogeneity, such as chromosome copy number variations, point mutations, genes rearrangements or epigenetic modifications that result in phenotypic diversity and *intercellular heterogeneity*. Interestingly, this intercellular heterogeneity, which may promote the development of new subclones, is empowered by genomic instability, which is in turn influenced by cancer treatment [1].

In human tumors, ITH has been documented by Software Inference methods able to infer evolutionary relationships between clonal subpopulations based on variant allele frequencies of point mutations and taking into account copy number alterations at the mutated loci [2,3]. According to these analysis, ITH is highly variable among tumors of different type. For instance, melanomas are highly polyclonal tumors (8-10 clones/tumor), whereas thyroid cancer (TC) has a mean low number of clones (2-4/tumor) [2].

Subclonal genetic alterations may be present only in a fraction of cells of distinct regions within the same tumor (*spatial heterogeneity)*, or subclonal genetic alterations of the primary tumor may be different from those of subsequent local or distant recurrences of the same patient (*temporal heterogeneity*) [4]. This phenomenon can be clinically relevant because the genetic pattern found in the primary tumor that in some cases steers the clinical and therapeutic decisions, may evolve during tumor progression, in particular in the regional or distant metastases, also due to the selection pressure of treatment [5]. Accordingly, a discordant pattern between the primary and the metastatic site has been recorded in several tumors (including lung, melanoma, colorectal, gastric, and breast), with a prevalence ranging 0%–49%. Some mutations are found to be private to the primary tumor, some private to metastases, and other shared [4,6].

ITH is likely to be the consequence of either the presence of two mutations in different clones in the primary tumor and to the distant propagation of only one clone, or to the acquisition of a secondary event at the metastatic site [7]. In this context, it would be critical to identify the cancer cells that have the potential to contribute to disease progression, in order to develop more effective cancer therapies.

In TC, two possible mechanisms underlying ITH were proposed. The hierarchical or cancer stem cell (CSC) model proposes that TC arises from the remnants of undifferentiated fetal thyroid cells, especially thyroid stem cells. According to the step of differentiation of the stem cell, after genetic and epigenetic transformations, phenotypically different cancer cells are generated with different degrees of aggressiveness [8,9,10,11]. Recently, sphere-growing cells were isolated from primary cultures of PTC, which fulfilled the definition of CSC and showed evidence of coordination in controlling tumorigenesis and progression [12].

The second mechanism is a paradigm of the carcinogenesis and the most accepted theory of thyroid tumor development, i.e., the stochastic multistep model, which is based on the theory of cancer clonal evolution [13]. This model states that tumor formation is a consequence of genome instability within somatic cells, which can lead to the appearance of more aggressive clones able to survive the selection pressure of the microenvironment and to outcompete other cells. This may result in a genetically homogenous tumor until a new more significant mutation appears. In this model, distinct molecular alterations were associated with specific stages that drive progression from well-differentiated to undifferentiated follicular-derived thyroid carcinomas [14]. A clear example of this process is the well demonstrated appearance of mutations, e.g., in TERT, PI3k or p53, in poorly differentiated thyroid carcinomas (PDTC) and in anaplastic tumors (ATC), which add themselves to pre-existing genetic alterations e.g., *BRAF* or *RAS* point mutations [15,16,17,18]. Consistently, the mutational burden is higher in less differentiated tumors than in differentiated or pediatric tumors [19], and associate with a more advanced stage and a worst prognosis [20]. Although the ITH developed during the process of dedifferentiation in PDTCs and ATCs is well established, the presence of ITH in the first phases of TC progression is a debated topic. The present review reports and discuss the data published in the literature related to the impact of ITH in the origin and progression of papillary thyroid cancer (PTC), which accounts for almost 80% of all TC cases, and to the diagnostic and clinical implications related to this phenomenon.

## 2. Intratumoral Heterogeneity in Papillary Thyroid Cancer

Papillary thyroid cancer is the most frequent endocrine tumor, and has in general a good prognosis, though a small fraction shows higher aggressiveness, and cannot be cured by standard treatments such as surgery and radioiodine. For these advanced cases, targeted therapies were recently developed directed towards either angiogenic pathways or genes known to be involved in thyroid carcinogenesis [21]. Somatic mutations in the mitogen-activated protein kinase (MAPK) pathway are found in nearly 70% of PTC, being *BRAF*^V600E^ the most frequent variant. Despite its potential clinical relevance, ITH was scantly investigated in PTC, probably due to the low number of oncogenes involved, which are frequently mutually exclusive, compared to other cancers [22], such as lung cancer and melanoma, where multiple oncogenes are frequently found to be altered in the same tumor at early stages [5]. As an example, subclonal *BRAF*^V600E^, the most frequent mutation in melanoma, was described in several reports. In particular, single cells genotyping showed *BRAF*^V600E^ heterogeneity within metastatic primary melanomas [23], in melanoma metastases [24], and among circulating tumor cells [25]. Similarly, subclonal *RAS* mutations were described in melanomas, and *N-RAS* and *BRAF* activating mutations were demonstrated to coexist in the same melanoma in different cells [26].

Unlike melanoma, PTC is considered to be largely homogeneous, so that a subtype classification was proposed according to the mutation detected, i.e., *BRAF-*like and *RAS-*like PTC [22]. Extended analysis of large series of tumors demonstrated a relatively low overall density of somatic mutations that is believed to be the biological basis for the indolent clinical behavior of PTC. Technical issues can partly explain the scanty data on ITH in PTC: the genetic pattern of PTCs was mostly investigated by the low sensitive sequencing Sanger method and was not to identify mutations present with a low allelic frequency [27]. Thyroid tumors consist of neoplastic cells intermingled irregularly with normal (connective tissue and vessels) and reactive (stromal and immune) cells, and the ratio between these components may vary largely between tumors [28]. Thus, the neoplastic cells content and the allelic frequency of the mutated oncogene in a given sample can be extremely low and below the sensitive threshold of many analytical methods. Accordingly, in PTC the allelic frequency must be normalized for the percentage of tumor cells in the sample, a measurement not always easy to perform [22,29]. Indeed, the number of the studies included in the present review which report data on the genetic characterization and/or ITH in TC, not evaluating tumor purity [27,30,31,32,33,34,35,36,37,38,39,40,41,42,43,44,45,46,47,48,49,50,51,52] are significantly more numerous than those which normalized the data for the percentage of tumor cells [7,20,22,29,53,54,55]. Thus, data on ITH obtained without considering the purity of the tumor must be considered with caution. Moreover, it should be underlined that a rigorous method to establish the genetic heterogeneity and clonality of cancer should include the evaluation of copy number alteration, too. Recent data from the pan genomic characterization of a synchronous FTC, PDTC and ATC showed that the cancer cells fraction determination (CCF), which denotes the proportion of cells among all cancer cells carrying a specific genetic aberrancy, allows precisely establishing the clonal composition of the tumors during the dedifferentiation process [19].

More recent methods of genetic analysis, i.e., pyrosequencing, allele-specific locked nucleic acid PCR, and next-generation sequencing, made possible a deeper and quantitative analysis of the mutational status of tumor samples, providing data in favor of ITH in PTC. Nevertheless, the existence and the relevance of subclonality in PTC is still debated since discordant evidence is available to date [56]. Moreover, the contribution of *intratumoral heterogeneity* to thyroid metastatic cancers and the clonal relationships between the primary thyroid tumor and lymph node or distant metastases are still unknown.

## 3. Evidence in Favor of ITH in PTC

Much evidence has been reported supporting the occurrence of ITH in PTC, either in early or advances stages of progression (Table 1).

### 3.1. Heterogeneous Presence of a Mutation Documented by Genetic Analysis

The first evidence of ITH in PTC came from studies evaluating the presence of *RET/PTC* rearrangements. The distribution of *RET* fusions was investigated by means of different approaches, demonstrating to vary in sporadic PTC or in post-Chernobyl PTC cases. The analysis of *RET* rearrangements by interphase fluorescence in situ hybridization (FISH) in 29 adult and 13 childhood post-Chernobyl PTCs unveiled that in all positive cases (23 and 10, respectively), the tumors were composed of a mixture of cells with and without *RET* rearrangements [30,31]. This ITH was further confirmed by a different research group that analyzed by FISH 14 *RET/PTC* positive PTC, finding nine cases with 50%–86% positive cells and five cases with 17%–35% positive cells [32]. High level of recombinant *RET/PTC* mRNA, a finding that the authors considered compatible with a clonal occurrence of the recombination, was observed only in 46% of *RET* rearrangements-positive adult PTC [33]. Interestingly, immunohistochemistry and reverse transcriptase-polymerase chain reaction (RT-PCR) analyses performed on RNA extracted after laser capture microdissection, and FISH experiments demonstrated the subclonal occurrence of *RET/PTC* rearrangements not only in PTC but also in hyperplastic or adenomatous nodule and even in scattered thyroid cells in Hashimoto’s thyroiditis [33,34]. In the study by Zhu et al. different detection methods with different sensitivity (standard-and high-sensitivity RT-PCR, real-time Light Cycler RT-PCR, Southern blot analysis, and FISH) demonstrated the subclonal or non-clonal occurrence of *RET/PTC-1* and *-2* in 17 of 65 (26%) PTC, while the clonal occurrence was demonstrated only in 9 (14%) tumors [32].

In following years, ITH of *BRAF*^V600E^ was demonstrated in PTC by different molecular techniques (Table 2). By pyrosequencing analysis, the subclonal or even oligoclonal occurrence of *BRAF*^V600E^ mutation was found to be more frequent than the clonal occurrence [36,57]. *BRAF*^V600E^ was demonstrated in more than 45% of alleles only in a minority of cases, indicating that *BRAF*^V600E^ mutation is frequently an oligoclonal event [57]. This data was confirmed by Gandolfi et al. [7] who showed by pyrosequencing in 58 *BRAF*^V600E^-positive PTC, an average mutated allele percentage of 27.44%, with a range between 7.50% and 49.80%. Further demonstration that both *BRAF*^V600E^ positive and negative cells can coexist in classic PTC was achieved by pyrosequencing of 264 manually microdissected *BRAF*^V600E^-positive tumors in which the mutant allelic frequency ranged 8%–41% of the total *BRAF* alleles (median, 20%) [37]. Based on these studies, for both *RET/PTC* and *BRAF*^V600E^ the subclonal occurrence in PTC appears to be something more than a rare event. The demonstration that the PTC tumor mass frequently consists of a mixture of few cells bearing mutant *BRAF* and more abundant tumor cells bearing wild-type *BRAF* was further confirmed after normalization for the percentage of tumor cells, and by the analysis of single cells obtained by laser capture [57]. De Biase et al. applied the allele-specific locked nucleic acid PCR to 155 PTC to determine the presence of *BRAF*^V600E^ and the allelic frequency after subtraction of non-tumor cells [29]. They observed that 10.6% PTCs displayed < 30% of *BRAF*^V600E^ neoplastic cells, and 45.9% PTCs displayed 30–80% of *BRAF*^V600E^ neoplastic cells. Overall, the 63.8% of PTCs had less than 80% of mutated cells. The heterogeneous distribution of *BRAF*^V600E^ in PTC, indicating subclonality or even oligoclonality, was confirmed in subsequent studies by means of next generation sequencing (NGS). The analysis of 30 *BRAF*^V600E^-positive PTC by 454 NGS, revealed a mean and median of *BRAF*^V600E^-positive neoplastic cells of 72.3% and 83%, respectively [29]. In another study, after the exclusion of non-tumoral cells by means of a morphometric analysis, 24% of 49 *BRAF*^V600E^ positive PTC were found to be subclonal by Ion Torrent-NGS [53]. These agreeing findings obtained in different laboratories by means of different methods, led to hypothesize that this mutation is not always the first transforming genetic event and that it can be a secondary event in PTC tumorigenesis [38,39]. More recently, the analysis of the ITH of PTC was extended to *TERT* and *RAS* genes. After normalization for tumoral cell content, MassARRAY genotyping confirmed the finding of the mutations in these two genes at low allelic frequencies in some samples, consistent with their presence in a small subset of cancer cells [20,24]. Recent data obtained by a multi-region WES approach on 257 PTC tumor tissues showed the presence of a subclonal driver alteration in 29% of tumors [58].

### 3.2. ITH of BRAF^V600E^ by Immunodetection 

A monoclonal *BRAF*^V600E^ mutation-specific antibody (VE1) was developed by Capper et al. in 2011 [66]. The heterogeneous staining described in some melanoma samples in the first report, was imputed to necrosis, though in subsequent studies this technical issue was no more reported and this antibody was considered a valid tool to investigate the intratumoral distribution of *BRAF*^V600E^ [66]. In a series of 85 PTCs analyzed by immunohistochemistry (IHC) with the VE1 antibody, 37 cases (43.5%) displayed more than 80% of stained cells, 39 cases had 30%–80% (45.9%), and nine had less than 30% (10.6%) stained cells [29]. In a different laboratory, when stained with the same antibody, immunoreactive and non-immunoreactive PTC cells were clustered separately or were intermingled in the primary lesions and in the corresponding metastatic lymph nodes [7]. These data indicated that as for the primary lesions, the matched lymph nodes where heterogeneous for the *BRAF*^V600E^mutation. Contrasting data come from Ghossein et al. who found a homogeneous staining in 13/14 PTCs, in 3/3 poorly differentiated TCs, and in 12/14 anaplastic TCs, supporting the concept that the *BRAF*^V600E^ mutation is a clonal event in the majority of TCs while ITH is a rare occurrence [67]. IHC with VE1 was employed in many studies to determine its reliability as diagnostic tool, though the issue of heterogeneity has not been addressed and, currently, the tumor is considered positive when a significant percentage of tumoral cells is stained. 

### 3.3. Presence of Concomitant Mutations

In recent years, also thanks to advances in NGS technology, it has become evident that multiple mutations can be concomitantly present in the same tumor [68,69]. In the context of PTC, co-occurrence of mutations, such as *BRAF*^V600E^, *TERT, RET/PTC* and *H4/PTEN* has been frequently documented, indicating that these genetic alterations might coexist in the same tumor [40,42,43,44,45,46,47]. Dual mutation of *BRAF*^V600E^ and *RET/PTC* and of *BRAF*^V600E^ and *TERT* promoter [37,48,53,54,57] and of point mutations and fusions [20] were found to occur in up to 20% PTCs. Although sporadically, concomitant occurrence of different *RAS* mutations or mutations of different *RAS* isoforms or concomitant *RAS* mutations and *RET/PTC* rearrangements were reported [49,50].

### 3.4. Discordant Mutational Status between Primary Site and Metastases

The multistep/multigene model with a progressive acquisition of new genetic defects is a paradigm of carcinogenesis. A discordant mutational status between the primary site and the metastases supports this mechanism and it was recorded in several human tumors (including lung, melanoma, colorectal, gastric, breast) [4]. In TC, discordant patterns were reported for *BRAF*^V600E^, *TERT, RAS* and other mutations [27,39,40,51,52,58,59,60,61,62,63,64,65]. Data related to PTC are reviewed in Table 3. In particular, the *BRAF* mutational status between primary tumor and metastases, mainly loco regional, was found to be discordant in up to 50% of cases, by different techniques [39,40,51,52,59,61,62]. In a recent study, it was shown that 14/27 TERT mutated primary tumors had wild-type *TERT* lymph node or distant metastases [52]. The loss in the metastases of the *BRAF* or *TERT* mutation present in the primary site is a very unlikely occurrence, hence these data strongly support the ITH of the primary tumors. Additional findings concerning ITH, and clonal relationship between primary tumor and metastases come from a case report of an aggressive PTC with matched lymphnode and a pleural metastasis. By the analysis of single nucleotide variants, gene fusions, and loss of heterozygosity, the authors showed that some of the genetic alterations were ubiquitously detected in all the tumor samples from the patient and others were detected only in some tumor areas, indicating the presence of several subclones in the neoplastic tissues. Interestingly, the two selected areas of the primary tumor and the two selected areas of on regional metastasis presented similar genetic profiles, whereas the two selected areas of another regional metastasis had more divergent mutations and fusions [55]. Striking heterogeneity was also observed between paired primary tumors and metastases in studies done by means of NGS and WES studies [58,60,63,64]. 

**Figure 1:**
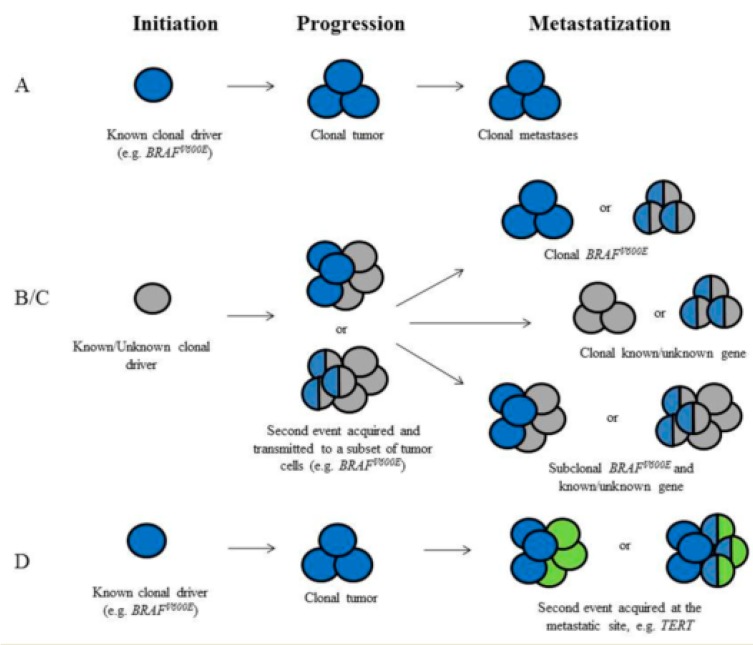
Hypotheses related to papillary thyroid cancer oncogenesis and progression (see text). (**A**) A known mutation occurs in the thyroid follicular cell and leads to the development of the tumor. (**B**,**C**) The tumor is established by a genetic driver (known or unknown). A second genetic event is acquired either in the same cell (**B**) or in a different cells (**C**) and transmitted with a sub-clonal distribution at the primary site. (**D**)A second genetic event is acquired at the metastatic site.

The contribution of ITH to thyroid metastatic cancers still needs to be more studied and clarified, though the finding of genetic heterogenous PTCs allows proposing hypotheses related to thyroid oncogenesis and progression (Figure 1).

(A) A known mutation occurs in the thyroid follicular cell and leads to the development of the tumor. Thereafter, the mutation is clonally distributed in all the tumor cells at the primary site and propagated to all the metastases developed during tumor progression. Primary tumor and metastases are clonal and the driver gene is detectable in both. This scenario is likely to be extremely frequent for PTC.

(B) The tumor is established by the transformation of a thyroid cell by a genetic driver (known or unknown). A second genetic event is acquired and transmitted to a subset of tumor cells at the primary site (sub-clonal distribution). The second genetic event can occur either in the same cell or in a different cell. This heterogeneous pattern can lead to the development of metastases with different genetic assets, namely cells with a double oncogenic expression or cells with monogenic expression intermingled with cells with a double oncogenic expression.

(C) A second genetic event is acquired at the metastatic site, either in the same cell or in different cells. The acquisition of a second event is predicted to increase the growth potential leading to the development of a clinically evident metastasis. 

## 4. Evidence Limiting the Impact of ITH In PTC

While large data support the IHT in PTC, its biological significance and clinical impact is debated. In some studies, the occurrence of ITH has been called into question or it is considered a limited or extremely limited phenomenon in PTC. With the Cancer Genome Atlas (TCGA) project, whole genome DNA of a large PTC cohort was examined by NGS [22]. This study reported somatic mutations in 83% and gene fusions in 13% of cases, mostly affecting the RAS/RAF/MAPK pathway. With very few exceptions, all mutations had an allelic frequency below 50%. However, subtracting the non-cancer cells estimated by a computational method that uses a pre-computed statistical models of recurrence cancer karyotypes [70], the major driver mutations documented (*BRAF*^V600E^, *RAS* mutations, and *RET/PTC*) were present in the majority of tumor cells with only very few exceptions. These findings led the authors to conclude that the tumors were largely clonal and that oligoclonality or polyclonality with respect to these oncogenes is a phenomenon limited to a few PTC cases. However, it is dutiful to highlight that FISH and PCR-based analysis are concordant assigning to ITH a significant impact in PTC, while NGS analysis are discordant. More recently, we analyzed a large cohort of 208 PTC by MassARRAY, and we calculated the allelic frequencies of *BRAF*^V600E^ and *RAS* mutations by subtracting non-tumor cells. The majority of cases had an allelic frequency of the mutated allele consistent with a monoclonal origin of the tumor, suggesting the occurrence of ITH in a small, though not negligible, subset of tumors (8%) [20].

## 5. Heterogeneity of Thyroid Cancer: Clinical and Therapeutic Implications

### 5.1. Spatial Heterogeneity

*Impact on clinical behavior*: some studies showed that mutation density highly correlates with aggressive histologic features and risk of recurrence [20,22]. Although the association of mutation density with worst outcome was not found in one study including cases with high-recurrence risk [71], most studies report that patients with dual mutations are associated with an older age at diagnosis and a worst outcome, suggesting that tumors with multiple mutations undergo a positive selection and are more aggressive [40,43,44,45,47]. Of note, *TERT* mutations were found to be mainly subclonal in PTCs, whereas they were clonal in poorly differentiated and anaplastic tumors, consistent with a positive selection during tumor evolution [54]. As far as *BRAF*^V600E^concerns, subclonal mutations were associated with smaller PTC tumors [29,37,53,57,72], lower extrathyroidal extension [37,53] and lower recurrence rate [57], while other studies did not report significant association with disease progression [7]. In contrast, Masoodi et al. showed that subclonal mutations were significantly higher in relapsed PTCs and that cases with high burden of subclonal mutations were associated with distant metastasis and increased risk of relapse or death [58].

*Impact on diagnosis*: as stated for other tumors [73], the presence of molecular heterogeneity highlights the importance of sampling multiple areas of the same tumor to better ascertain the range of genomic alterations characterizing its progression. In a recent study, it was found that the 23.1% of the somatic mutations found in a large PTC series were not identified in all regions of the tumor, revealing that ITH limits the diagnostic value of single diagnostic biopsy sample [58]. Particularly in advanced TCs, the heterogeneous presence of genomic alterations may impair patient genotyping and subsequent prognostic classification and targeted therapy. Moreover, some mutations, despite their very low allelic frequencies in the primary tumor, can be responsible for the development of metastases, indicating the need for highly sensitive diagnostic tools to obtain a full genetic characterization.

*Impact on treatment*: ITH could have a significant impact on prognosis and treatment response and may influence the best and targeted therapeutic strategy, especially in the era of personalized medicine [5]. Indeed, the genetic pattern found in the primary tumor that in some cases directed the clinical and therapeutic decisions, may be not representative of the variation within a tumor as a whole, and may evolve during tumor progression, also due to the selection pressure of treatment [4]. Consequently, for many tumors, combinations of targets-based drugs will likely be necessary to control the tumor growth.

### 5.2. Temporal Heterogeneity

The different subclones are predicted to have different resistance mechanisms to treatment. If they are all sensitive to the initial treatment, the tumor will be eradicated. Cancer relapse can be due to the progressive enrichment with time of drug resistant cancer cells already present in the heterogeneous cancer cell population. Indeed, it is predicted that a fraction of cells with stem cell properties and probably also a fraction of adult cells within the heterogeneous population are drug resistant, able to survive to treatment and expanding over time [74], as shown for some cancers such as lung [75]. It is worth mentioning that temporal heterogeneity derives also from the progressive increase of the mutational burden during the de-differentiation process [15,16,17,18]. The best way to identify the presence of different subclones, is to submit the primary tumor to an ultra-deep sequence which allows identifying all the mutated clones, in order to start a treatment directed by the characteristics of the dominant clone as well as the rare resistance clones, with a combination of therapies to eradicate all clones. Nevertheless, this option is rarely applied, especially for TC which is a tumor well curable in the vast majority of cases.

Interesting insights come from a recent report of an acquired *KRAS* mutation that developed during treatment with BRAF and MEK inhibition in a patient with a *BRAF*-mutated PTC. The *KRAS* mutation was detected at the time of progression in the peripheral blood, too [76]. This last finding demonstrates that resistant mutations can be documented even with non-invasive methods, such as plasma DNA or circulating tumor cells [77].

Additional tools to identify resistant subclones, imply their analysis in cellular systems, which also allows the testing for different and possible novel therapeutic options. As far as TC concerns, Antonello et al. expanded a sub-population of cells with primary resistance to vemurafenib and found that they harbor amplification of chromosome 5 and mutations in *RBM* genes which are crucial for genome stability during cell division [78]. A combined therapeutic approach using BRAF^V600E^ and CDK4/6 inhibitors was able to induce apoptosis in both naïve and vemurafenib-resistant cells, indicating that this combined therapy could be tested in a clinical trial of advanced TC patients. 

## 6. Conclusions

Intratumoral genetic heterogeneity identify a phenomenon by which a neoplasm harbors genetically different subclones that may be intermixed or spatially separated. Importantly, the number of genomic alterations spontaneously increases with tumor progression and can evolve in response to treatments. Different types of cancer are characterized by different subclonal complexity, sometimes with a high number of subclones such as melanoma, and other times showing a low number such as the case of thyroid cancer. The existence of the ITH is well accepted for advanced PTC, while it is a debated issue for PTCs in the early stage of progression. Several data presented in this review indicate that ITH is not a marginal event that can occur in PTC at any step of development, including early stage PTC. Furthermore, the demonstration of ITH of *BRAF* and *RAS* mutations and *RET/PTC* rearrangements highlights the existence of unknown genetic or epigenetic alterations that still need to be identified.

The ITH of *BRAF* and *RAS* mutants and *RET/PTC* rearrangements in early stage PTC, call into question their role of driver mutations and initiators of thyroid carcinogenesis. In a stochastic model of multi-step carcinogenesis, the coexistence of *BRAF* mutation negative and positive subclones entails that these oncogenes are generated succeeding other unidentified genetic alterations which have the role of initiators. In the cancer stem cell (CSC) model, a small subpopulation of CSCs that can self-renew and differentiate to produce phenotypically diverse cancer cells acquires initiating mutations, with *BRAF*, *RAS* and *RET/PTC* alterations developing later, and involving only a cellular subpopulation. 

Based on the present knowledge of this topic, the definition of the TC genetic background must consider the existence of either heterogeneity or multiple mutations with different allelic percentage. In this context, benefits will come from NGS techniques (especially whole genome sequencing) since these tools allow better appreciating clonal cancer cell fractions of important mutations mostly because of the high-resolution CNV analyses. It is also well known that the presence of intra-tumor heterogeneity is the cause for relapsing after the therapy, which effectively eliminated the competition of the major clone, leaving minor subclones free to expand. The assessment of such complexity, for instance by the analysis of paired primary and metastatic samples from the same patient to acquire insights into clonality and subclonality patterns of genomic events, is mandatory for the further development of personalized medicine in TC.

Since novel generation compounds highly selective for a specific genetic alteration are currently on trial, the assessment of the genetic pattern of malignant tumors, including advanced TC, is definitely needed. The genetic evaluation should consider the identification of the percentage of mutated cells, too, by normalization for the non-tumoral cell content of the neoplastic mass, since it is predictable that different allelic frequencies of a given mutation could correlate with the response to treatment.

## Figures and Tables

**Table 1:** Arguments in favor and in contra on the presence of extensive intratumoral heterogeneity (ITH) in papillary thyroid cancer.

Proofs of Extensive ITH	Ref.
(1) Heterogeneous presence of a mutation documented by genetic analysis	Fusco et al. 2002 [34], Unger et al. 2004 [30], Vasko et al. 2005 [39], Unger et al., 2006 [31], Zhu et al. 2006 [32], Rhoden et al. 2006 [33], Hieber et al. 2011 [35], Guerra et al. 2012 [36], Guerra et al. 2012 [57], Xing et al. 2012 [38], Gandolfi et al. 2013 [7], De Biase et al. 2014 [29], Kim et al. 2014 [37], Muzza et al. 2015 [40], Finkel et al. 2016 [53], Colombo et al. 2019 [20], Masoodi et al. 2019 [58]
(2) Heterogeneous presence of BRAF^V600E^ by immunodetection	Gandolfi et al. 2013 [7], De Biase et al. 2014 [29], Dvorak et al. 2014 [41]
(3) Presence of concomitant mutations	Sugg et al. 1999 [50], Wang et al. 2008 [42], Henderson et al. 2009 [45], Guerra et al. 2012 [57], Landa et al. 2013 [46], Kim et al. 2014 [37], Xing et al. 2014 [43], Guerra et al. 2014 [44], Wang et al. 2014 [47], Muzza et al. 2015 [40], Shrestha et al. 2015 [48], Rossi et al. 2015 [49], Finkel et al. 2016 [53], Landa et al. 2016 [54], Colombo et al. 2019 [20], Masoodi et al. 2019 [58]
(4) Discordant mutational status between primary site and metastases	Vasko et al. 2005 [39], Oler et al. 2005 [59], Ricarte-Filho et al. 2009 [27], Walts et al. 2014 [51], Muzza et al. 2015 [40], Le Pennec et al. 2015 [55], Sohn et al. 2016 [60], Caňadas-Garre et al. 2016 [61], Melo et al. 2017 [52], Fakhruddin et al. 2017 [62], Shifrin et al. 2017 [63], Masoodi et al. 2019 [58], Masoodi et al. 2019 [64], Gawin et al. 2019 [65]
Cons of extensive ITH	
(1) Heterogeneous presence of a mutation documented by genetic analysis only in a minority of cases	Cancer Genome Atlas Network 2014 [22], Colombo et al. 2019 [20], Masoodi et al. 2019 [58]

**Table 2:** Studies reporting significant BRAFV600E intratumoral heterogeneity by different techniques.

Technique	BRAF^V600E^ Clonal Status
**VE1 Anti-BRAF^V600E^ antibody**	Stained and non-stained PTC cells clustered separately or intermingled in the primary and in the metastatic lymph nodes. Gandolfi et al. 2013, [7].
	36% of PTCs displayed < 80% of *BRAF*^V600E^ positive cells. De Biase et al. 2014, [29].
	Unspecified percentage of PTC displayed < 15% stained cells. Dvorak et al. 2014, [41]
**Pyrosequencing**	66.5% of *BRAF*^V600E^ PTCs presented an allelic frequency ranging 5.1%–25%. Guerra et al. 2012, [57]
	54.9% mean of cells with *BRAF*^V600E^ in mutation positive PTC. Gandolfi et al. 2013, [7]
	20% median of cells with *BRAF*^V600E^ in mutation positive PTC. Kim et al. 2014, [37]
	19.4% PTCs had a dual mutation *BRAF*^V600E^ and *RET/PTC*, with a *BRAF* allelic frequency ranging 6–37.5%. Guerra et al. 2014, [44]
**Allele-specific locked nucleic acid PCR**	10.6% PTCs with < 30% of *BRAF*^V600E^ neoplastic cells; 45.9% PTCs with 30%–80% of *BRAF*^V600E^
	neoplastic cells. De Biase et al. 2014, [29]
**Next Generation Sequencing**	72.3% mean of cells with *BRAF*^V600E^ in mutation positive PTC. De Biase et al. 2014, [29]
	24% of the *BRAF*^V600E^ PTCs were subclonal. Finkel et al. 2016, [53]
	*BRAF*^V600E^ was subclonal in 30% primary non-relapse and in 44% primary relapse PTC. Masoodi et al. 2019, [58]
**Sequenom MassArray**	12.6% of *BRAF*^V600E^ PTCs had an allelic frequency lower than 50%. Colombo et al. 2019, [20]

**Table 3:** Mutational status of metastatic sites with respect to corresponding primary papillary thyroid cancer.

Genetic Analysis	Metastatic Site	Mutational Status with Respect to Primary Tumor	Ref.
BRAF exon 15 direct sequencing and single-strand conformational polymorphism	lymph node	9 concordant/4 discordant	Oler et al. 2005 [59]
BRAF exon 15 direct sequencing	lymph node	26 concordant/7 discordant	Vasko et al. 2005 [39]
BRAF exon 15 and TERT promoter mutation direct sequencing	lymph node	9 concordant/3 discordant	Muzza et al. 2015 [40]
BRAF exon 15 direct sequencing	lymph node	23 concordant/2 discordant	Walts et al. 2014 [51]
Target NGS, TERT promoter mutation direct sequencing, qRT-PCR of RET and PAX8 fusions	lung brain	5 concordant/2 discordant 2 concordant	Sohn et al. 2016 [60]
BRAF exon 15 and N-RAS exon 2 and 3 direct sequencing	lymph node and distant metastases	14 concordant/15 discordant	Caňadas-Garre et al. 2016 [61]
BRAF, K- and N-RAS mutations by reverse hybridization	lymph node	38 concordant/5 discordant	Fakhruddinet et al. 2017 [62]
Target NGS	lymph node	13 concordant/8 discordant	Shifrin et al. 2017 [63]
BRAF exon 15, N-RAS exon 2 and 3, TERT promoter mutation direct sequencing	lymph node and distant metastases	almost concordant discordant	Melo et al. 2017 [52]
WES	bone lung brain kidney	7 concordant/2 discordant * 2 concordant/1 discordant * 1 discordant * 1 discordant *	Masoodi et al. 2019 [64]
WES	local relapse	4 concordant/1 discordant	Masoodi et al. 2019 [58]
Mass spectrometry imaging	lymph node	discordant	Gawin et al. 2019 [65]

Legend: * for likely pathogenic cancer genes.
